# Occlusion of a Vortex Vein After Treatment With Half-Fluence Photodynamic Therapy Combined With Intravitreal Aflibercept Injection for Pachychoroid Neovasculopathy

**DOI:** 10.7759/cureus.27663

**Published:** 2022-08-04

**Authors:** Chizu Yamada, Ryo Mukai, Yoichiro Shinohara, Hidetaka Matsumoto, Hideo Akiyama

**Affiliations:** 1 Department of Ophthalmology, Gunma University Graduate School of Medicine, Maebashi, JPN

**Keywords:** aflibercept, large vessel occlusion, vortex vein occlusion, pachychoroid neovasculopathy, photodynamic therapy

## Abstract

Photodynamic therapy (PDT) is a treatment option for pachychoroid diseases such as central serous chorioretinopathy (CSC), pachychoroid neovasculopathy (PNV), polypoidal choroidal vasculopathy (PCV), and peripapillary pachychoroid syndrome (PPS). On the other hand, morphological changes of choroidal vessels in the irradiated field after PDT have also been discussed, with occlusion of choriocapillaris and stenosis of choroidal middle and large vessels being reported. Here, we report a case of vortex vein occlusion after half-fluence PDT (HF-PDT) combined with an anti-vascular endothelial growth factor (VEGF) agent for PNV. In this case, HF-PDT achieved complete occlusion of PNV; in addition, a vortex vein that flowed in PNV but was located outside the PDT irradiation field was fully occluded three months post-treatment. At the occluded site of the vortex vein, indocyanine green video angiography revealed pulsation downstream of the vortex vein. Such occlusion of a large vessel by HF-PDT has not been reported previously. Occlusion could be induced by two factors: the potentiality of PDT and risk factors for thromboembolism, such as older age, smoking, and arrhythmia. Further studies are required to determine the mechanisms of these large vessel occlusions.

## Introduction

Pachychoroid disease is a disease concept that describes a phenotype characterized by an attenuation of the choriocapillaris overlying dilated choroidal veins and is associated with retinal pigment epithelial dysfunction and neovascularization [1]. Central serous chorioretinopathy (CSC), pachychoroid pigment epitheliopathy (PPE), pachychoroid neovasculopathy (PNV), polypoidal choroidal vasculopathy (PCV), focal choroidal excavation (FCE), and peripapillary pachychoroid syndrome (PPS) reside within the pachychoroid disease spectrum [1].

PNV is characterized by type 1 macular neovascularization (MNV) in eyes with pachychoroid features. To distinguish PNV from neovascular age-related macular degeneration (nAMD), the current diagnostic criteria for PNV can be summarized as (1) the presence of pachychoroid features and (2) the absence of drusen [2].

Currently, anti-vascular endothelial growth factor (VEGF) therapy is the gold standard for nAMD, and its efficacy for PNV has been reported [3-5]. However, more extensive injections of anti-VEGF compared to PCV may be required to treat PNV. Photodynamic therapy (PDT) is one of the treatment options for not only CSC but also nAMD; PDT therapy can regress MNV and reduce vascular permeability of the choriocapillaris and choroidal thickness, which can contribute to the absorption of retinal fluid [6]. PDT combined with anti-VEGF agents appears to be a more potent tool for PCV treatment. The endovascular valve edge-to-edge repair study (EVEREST) II trial [7] revealed that the combination therapy of PDT and ranibizumab for PCV was superior to ranibizumab alone with respect to improvement of visual acuity and frequencies of polyp-regression. Recently, half-fluence PDT (HF-PDT) combined with anti-VEGF agents was also applied to patients with PNV to stabilize MNV and the choroid [8].

After PDT treatment, a circumscribed occlusion of the choriocapillaris was identified in the area where PDT was exposed using indocyanine green angiography (IA) [9]. In this study, eyes were surgically removed seven days after PDT, and a histological study of the PDT exposed area also revealed an occluded choriocapillaris filled with emboli, which was accompanied by deformed erythrocytes, degranulated platelets, and fibrin. These results suggest that the essential effect of PDT is the clogging of capillary vessels in the choroid.

In this case presentation, we present an unusual case in which a large vortex vein was occluded after HF-PDT with aflibercept intravitreal injection in a patient with PNV.

## Case presentation

**Figure 1:**
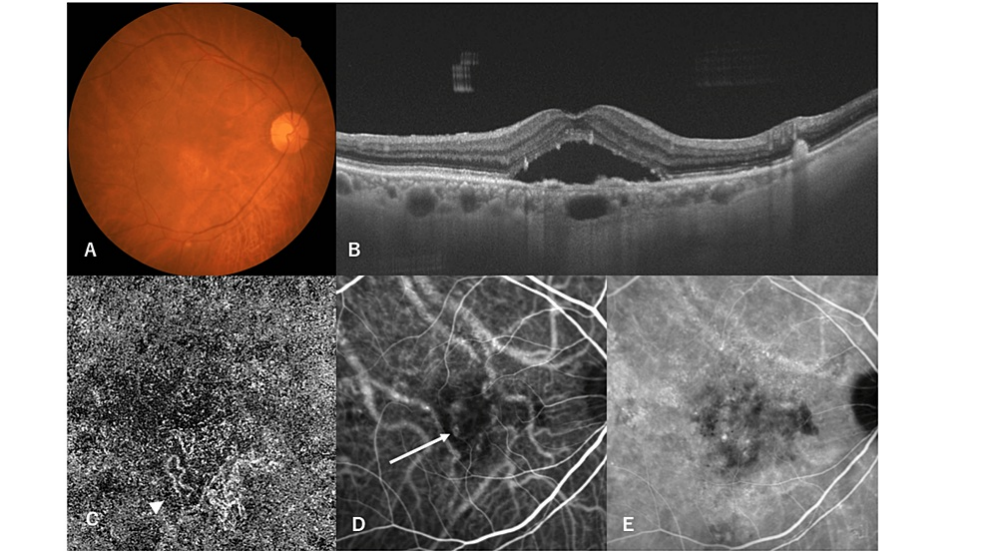
Multimodal imaging of the patient. (A) Color fundus photograph of the right eye shows retinal pigment epithelium (RPE) abnormality in the macular area. (B) Twelve-millimeter vertical B-mode optical coherence tomography (OCT) images through the fovea show the pachychoroid with dilated outer choroid vessels. A shallow irregular RPE detachment accompanied by serous retinal detachment is observed at the fovea. (C) OCT angiography shows network vessels of choroidal neovascularization between the detached RPE and Bruch’s membrane (arrowhead). (D) Indocyanine green angiography in the early phase shows a hyperfluorescent plaque overlying a large caliber choroidal vessel (arrow). (E) Indocyanine green angiography in the late phase shows choroidal vascular hyperpermeability in the area surrounding the neovascular tissue.

**Figure 2:**
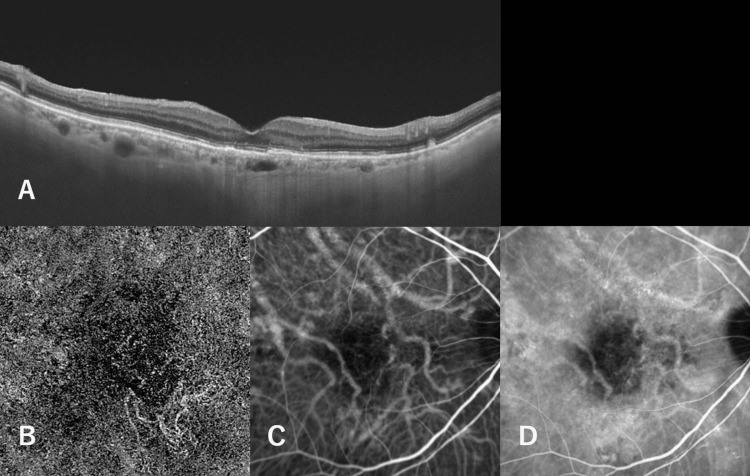
Three months after half-fluence photodynamic therapy (HF-PDT) combined with intravitreal aflibercept injection. (A) Vertical B-mode optical coherence tomography (OCT) through the fovea shows no serous retinal detachment, decreased choroidal thickness, and pigment epithelial detachment, which corresponded to PNV. (B) OCT angiographic image shows shrinkage of the capillary network of the neovascular vessels. (C) Indocyanine green angiography in the early phase shows regression of neovascularization. (D) Indocyanine green angiography in the late phase shows a hypofluorescent area where HF-PDT was applied.

An 89-year-old man was referred to our hospital because of impaired vision in the right eye. He had a medical history of arrhythmia (not medicated) and benign prostatic hyperplasia. His smoking history was 12 cigarettes per day for 30 years (from the age of 20 to 50 years). Best-corrected visual acuity was 20/32 in the right eye and 20/20 in the left eye. Optical coherence tomography (OCT) revealed serous retinal detachment accompanied by flat retinal pigment epithelial detachment in the right macula, in addition to a thickened choroid-associated dilatation of outer choroidal vessels in the same eye (Figure 1B). OCT angiography (OCTA) revealed choroidal neovascularization beneath the pigment epithelial detachment (Figure 1C). IA also identified choroidal neovascularization in the same area as OCTA, and dilated vortex veins adjacent to the CNV were detected (Figure 1D). Choroidal vascular hyperpermeability was observed in the late stage of IA. We diagnosed PNV and performed reduced fluence PDT (RF-PDT) with intravitreal aflibercept injection. Three months after treatment, the serous retinal detachment disappeared, and choroidal thickening decreased (Figure 2A). The CNV was successfully regressed and reduced in both IA and OCTA. IA was used to detect a circumscribed hypofluorescent area where HF-PDT was applied (Figure 2B, 2C).

**Figure 3:**
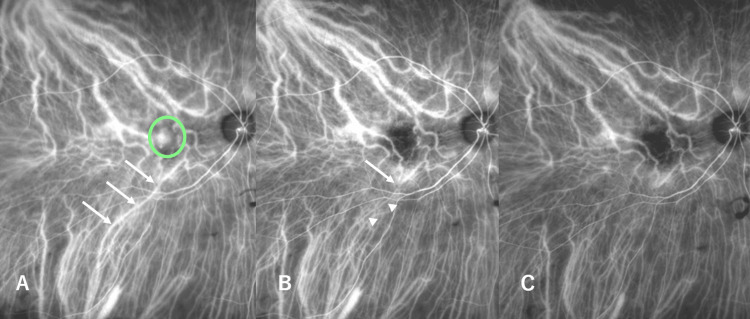
Indocyanine green angiography (IA) images describing vortex veins. (A) In the pre-treatment IA, a dilated vortex vein was observed (arrow). The area circled in green is the photodynamic therapy-treated field. (B) In IA, at three months post-treatment, there was an interruption in the vortex vein (arrow), and the downstream blood vessels were thinning (arrowhead). (C) At 14 months post-treatment, this vortex vein was completely occluded.

Three months post-treatment, IA also revealed occlusion of a vortex vein that branched in the inferior posterior region, outside the irradiated area (Figure 3A, 3B). A complete interruption in the vortex vein was observed without a downstream flow of the vessel in a movie of the IA (Heidelberg Engineering, Heidelberg, Germany) (Video 1). Interestingly, pulsation of the vortex vein at this portion was also detected, and the blood seemed to stream inversely when compared to the bloodstream at the initial visit. Fourteen months post-treatment, the IA movie revealed complete occlusion of the vortex vein, with no recanalization and no pulsation (Figure 3C).

**Video 1:** A vortex vein that flowed in PNV but was located outside the PDT irradiation field was fully occluded three months post-treatment.

Fortunately, no recurrence of MNV developed, the patient did not complain of any changes in vision during the follow-up period, and his final visual acuity remained unchanged at 20/32.

## Discussion

This case suggests that PDT can cause not only clotting of capillary vessels but also occlusion of large choroidal vessels.

Vascular occlusion at the level of the choroidal capillary plate after PDT has been reported previously [9,10]; however, occlusion of large vessels, as observed in this study, has not been reported before. 

Previous studies have identified that verteporfin is essentially taken up by abnormal neovascularization, leading to selective cytotoxicity of vascular endothelial cells through the production of oxidative radicals. This reactivity can cause regression of neovascularization and resolution of the leakage from the neovascular membrane [11-13].

On the other hand, PDT also affected normal choroidal vessels, specifically both normal choriocapillaris and middle and large choroidal vessels [10,14,15]. As mentioned in the introduction, the circumscribed hypofluorescent area where the PDT had been exposed was detected in IA after PDT treatment, and the area contained both normal and abnormal choroidal vessels [9]. OCT image analysis revealed thinning of the choroid after PDT treatment [16], and the vascular density in both the choriocapillaris and the middle layer of the choroid significantly decreased after the treatment. Moreover, the maximum vessel diameter in the outer choroidal layer in the area exposed to PDT was significantly reduced but not occluded [9]. In this case, occlusion of the large vortex vein outside the irradiated area could have been induced by three factors: the potentiality of PDT, the existence of a vessel branch traversing the irradiated area, which could have caused thromboembolism to the distal part, and risk factors for thromboembolism, such as older age, smoking, and arrhythmia [17].

On the other hand, indocyanine green angiography in the early phase showed hyperfluorescent plaque overlying a large caliber choroidal vessel in Figure 1D, which possibly corresponded to an anastomotic vessel connecting the upper and lower vortex veins [18]. In this case, the superior and inferior vortex veins were asymmetric. They seemed to be connected by anastomosis at the horizontal watershed zone. The superior vortex vein had a larger diameter, and the inferior vortex vein had a thinner diameter. Thus, the superior vortex vein should be responsible for compensatory venous flow before treatment. After treatment, blood flow seemed to return upward in a V-shape due to the trunk occlusion. The trunk of the inferior vortex vein could be no longer needed and considered to have been occluded by disuse. 

With the detection of the occlusive vortex vein, a pulsation was detected downstream of the occlusive portion in the IA. Pulsation of the retinal artery was previously reported in cases with central retinal vein occlusion, and the authors concluded that pulsation meant a delay in the retinal bloodstream. In pachychoroid spectrum diseases, pulsation in the choroidal large vasculature has also been detected in treatment-naïve cases [19]. These results suggest that choroidal overload might be associated with the disturbance of choroidal circulation. In this case, the backflow of the vortex vein downstream of the occlusive portion may have led to turbulent flow in this area. 

## Conclusions

To the best of our knowledge, we report a first case in which a vortex vein located outside the irradiated area was occluded after HF-PDT combined with intravitreal aflibercept. At the occluded site of the vortex vein, indocyanine green video angiography revealed pulsation downstream of the vortex vein. Pulsation on IA can be used as a biomarker to suggest an overload of choroidal circulation.

## References

[1] Cheung CM, Lee WK, Koizumi H, Dansingani K, Lai TY, Freund KB (2019). Pachychoroid disease. Eye (Lond).

[2] Yanagi Y (2020). Pachychoroid disease: a new perspective on exudative maculopathy. Jpn J Ophthalmol.

[3] Sartini F, Figus M, Casini G, Nardi M, Posarelli C (2020). Pachychoroid neovasculopathy: a type-1 choroidal neovascularization belonging to the pachychoroid spectrum-pathogenesis, imaging and available treatment options. Int Ophthalmol.

[4] Schworm B, Luft N, Keidel LF (2020). Ranibizumab non-response in pachychoroid neovasculopathy: Effects of switching to aflibercept. Sci Rep.

[5] Matsumoto H, Hiroe T, Morimoto M, Mimura K, Ito A, Akiyama H (2018). Efficacy of treat-and-extend regimen with aflibercept for pachychoroid neovasculopathy and Type 1 neovascular age-related macular degeneration. Jpn J Ophthalmol.

[6] Hata M, Tagawa M, Oishi A (2019). Efficacy of photodynamic therapy for polypoidal choroidal vasculopathy associated with and without pachychoroid phenotypes. Ophthalmol Retina.

[7] Koh A, Lai TY, Takahashi K (2017). Efficacy and safety of ranibizumab with or without verteporfin photodynamic therapy for polypoidal choroidal vasculopathy: A randomized clinical trial. JAMA Ophthalmol.

[8] Matsumoto H, Mukai R, Kikuchi Y, Morimoto M, Akiyama H (2020). One-year outcomes of half-fluence photodynamic therapy combined with intravitreal injection of aflibercept for pachychoroid neovasculopathy without polypoidal lesions. Jpn J Ophthalmol.

[9] Schmidt-Erfurth U, Schlötzer-Schrehard U, Cursiefen C, Michels S, Beckendorf A, Naumann GO (2003). Influence of photodynamic therapy on expression of vascular endothelial growth factor (VEGF), VEGF receptor 3, and pigment epithelium-derived factor. Invest Ophthalmol Vis Sci.

[10] Mukai R, Matsumoto H, Miyakubo T, Akiyama H (2021). Reduced vascular density in the choroid after treatment with photodynamic therapy combined with aflibercept in patients with polypoidal choroidal vasculopathy. Retina.

[11] Treatment of Age-Related Macular Degeneration with Photodynamic Therapy (TAP) Study Group (1999). Photodynamic therapy of subfoveal choroidal neovascularization in age-related macular degeneration with verteporfin: one-year results of 2 randomized clinical trials - TAP Report 1. Arch Ophthalmol.

[12] Treatment of Age-Related Macular Degeneration with Photodynamic Therapy (TAP) Study Group (2001). Photodynamic therapy of subfoveal choroidal neovascularization in age-related macular degeneration with verteporfin: two-year results of 2 randomized clinical trials - TAP Report 2. Arch Ophthalmol.

[13] Bressler NM, Arnold J, Benchaboune M (2002). Verteporfin therapy of subfoveal choroidal neovascularization in patients with age-related macular degeneration: additional information regarding baseline lesion composition's impact on vision outcomes-TAP report No. 3. Arch Ophthalmol.

[14] Demirel S, Özcan G, Yanık Ö, Batıoğlu F, Özmert E (2019). Vascular and structural alterations of the choroid evaluated by optical coherence tomography angiography and optical coherence tomography after half-fluence photodynamic therapy in chronic central serous chorioretinopathy. Graefes Arch Clin Exp Ophthalmol.

[15] Iovino C, Au A, Chhablani J (2020). Choroidal anatomic alterations after photodynamic therapy for chronic central serous chorioretinopathy: A multicenter study. Am J Ophthalmol.

[16] Maruko I, Iida T, Oyamada H, Sugano Y, Ojima A, Sekiryu T (2013). Choroidal thickness changes after intravitreal ranibizumab and photodynamic therapy in recurrent polypoidal choroidal vasculopathy. Am J Ophthalmol.

[17] Crous-Bou M, Harrington LB, Kabrhel C (2016). Environmental and genetic risk factors associated with venous thromboembolism. Semin Thromb Hemost.

[18] Matsumoto H, Kishi S, Mukai R, Akiyama H (2019). Remodeling of macular vortex veins in pachychoroid neovasculopathy. Sci Rep.

[19] Paques M, Baillart O, Genevois O, Gaudric A, Lévy BI, Sahel J (2005). Systolodiastolic variations of blood flow during central retinal vein occlusion: exploration by dynamic angiography. Br J Ophthalmol.

